# The Effect of Older Adults’ Relational Stress, Environmental Stress, and Coping on Well-Being

**DOI:** 10.1093/geroni/igaa057.2169

**Published:** 2020-12-16

**Authors:** Meeryoung Kim

**Affiliations:** Daegu University, Gyeongsan, Republic of Korea

## Abstract

This study used the stress process model for analyzing older adults’ stressors and coping resources, which compares relational and environmental stressors (Pearlin et al., 1990). Additionally, the effect of coping abilities and social support on well-being was compared. This study used the 5th wave data of KReIS (Korean Retirement and Income Studies) that were collected in 2014. The sample included 4,072 older Korean adults aged 60 and older. Relational and environmental stressors were used as the independent variables. Social support and coping were used as coping resources. For the dependent variable, life satisfaction and perceived health were used. Since the stress model is a process model, hierarchical multiple regression was used. Environmental stressors had a significant effect on reducing life satisfaction. Relational and environmental stressors had significant negative effects on perceived health. Both coping and social support had a significant effect on both life satisfaction and perceived health.

